# Brief Mobile App–Based Mindfulness Intervention for Indonesian Senior High School Teachers: Protocol for a Pilot Randomized Controlled Trial

**DOI:** 10.2196/56693

**Published:** 2024-10-23

**Authors:** Gede Rasben Dantes, Nice Maylani Asril, Andrian Liem, Ni Komang Arie Suwastini, Shian-Ling Keng, Ni Wayan Surya Mahayanti

**Affiliations:** 1 Department of Computer Science Faculty of Engineering and Vocational Universitas Pendidikan Ganesha Singaraja Indonesia; 2 Faculty of Education Universitas Pendidikan Ganesha Singaraja, Bali Indonesia; 3 Jeffrey Cheah School of Medicine and Health Sciences Monash University Malaysia Bandar Sunway Malaysia; 4 Faculty of Language and Art Universitas Pendidikan Ganesha Singaraja Indonesia; 5 Department of Psychology School of Medical and Life Sciences Sunway University Malaysia Bandar Sunway Malaysia

**Keywords:** digital mental health, telemedicine, anxiety, stress, self-efficacy, life satisfaction, self-compassion, mindfulness, feasibility study, mobile app, mindfulness-based stress reduction, stress management

## Abstract

**Background:**

The COVID-19 pandemic has increased the level of anxiety among Indonesian senior high school teachers, who face challenges to treat their mental disorder symptoms that arise during their working hours, as mental health services in Indonesia are limited. Therefore, it is vital to equip schoolteachers in Indonesia with early interventions that are easily available, private, and affordable, and 1 feasible approach is to deploy a smartphone mobile app.

**Objective:**

The objectives of this study are (1) to evaluate the feasibility of a brief mindfulness–based mobile app (BM-MA) for Indonesian senior high school teachers experiencing anxiety and stress and (2) to examine the effects of using the BM-MA on anxiety, stress, life satisfaction, self-efficacy, trait mindfulness, self-compassion, and physical and social dysfunction among the participants.

**Methods:**

We followed the SPIRIT (Standard Protocol Items: Recommendations for Interventional Trials) 2013 statement for this feasibility randomized controlled trial (RCT) protocol. A total of 60 Indonesian senior high school teachers were recruited for this study and randomly assigned to either the intervention group (BM-MA) or a wait-list control group (CG) in a 1:1 ratio. The BM-MA group was required to engage in mindfulness practices using the app for 10-20 minutes per day for 3 weeks. All participants were assessed with a battery of self-report measures at baseline, postintervention, and at 1-month follow-up. Validated scales used to measure the outcome variables of interest included the Satisfaction With Life Scale (SLS), the Teachers’ Sense of Efficacy Scale (TSES), the Self-Compassion Scale—Short Form (SCS-SF), Generalized Anxiety Disorder-7 (GAD-7), General Health Questionnaire-12 (GHQ-12), and the Five Facet Mindfulness Questionnaire (FFMQ). The practicality and acceptability of the app will be evaluated using the Client Satisfaction Questionnaire-8 (CSQ-8) and structured qualitative interviews. Data from the interviews will be analyzed with the deductive thematic analysis framework as a process of qualitative inquiry. Repeated measures ANOVA with groups (intervention vs control) as a between-subject factor and time as a within-subject factor (baseline, postintervention, and 1-month follow-up) will be used to examine the effects of the BM-MA on the outcome variables. The data will be analyzed using an intent-to-treat approach and published in accordance with CONSORT (Consolidated Standards of Reporting Trials) recommendations.

**Results:**

Participants were recruited in December 2023, and this pilot RCT was conducted from January through March 2024. Data analysis was conducted from March through May 2024. The results of this study are expected to be published in December 2024. The trial registration of this protocol was submitted to the Chinese Clinical Trial Registry.

**Conclusions:**

This study aims to determine the feasibility and efficacy of the BM-MA, a digital mental health intervention developed using an existing mindfulness-based app, and assess its potential for widespread use.

**Trial Registration:**

Chinese Clinical Trial Registry ChiCTR2300068085; https://tinyurl.com/2d2x4bxk

**International Registered Report Identifier (IRRID):**

DERR1-10.2196/56693

## Introduction

### Background

During the COVID-19 epidemic, 79.1% of government senior high school teachers in Jakarta, Indonesia, experienced stress due to workload, work engagement, work routine, and the workplace environment [1]. The higher stress level of schoolteachers during the pandemic was closely tied to the increased workload caused by a shift in the teaching format [2]. The educational strategy during the COVID-19 pandemic was ambiguous, leading to differences in how teaching was implemented. Teachers often interpreted the policy based on their existing knowledge and teaching skills [3]. Another ambiguity in the online school period was that teachers perceived a lack of explicit consequences, whether positive or negative, for the quality of their online teaching delivery [3,4].

Teachers between the ages of 36 and 45 years were found to experience greater levels of stress and anxiety than older teachers, since younger teachers were required to perform more technology-related duties that the older teachers could not perform [5]. Malini [5] also reported that schoolteachers lack a means of coping with their mental health. The condition of schoolteachers does not improve, and they receive less assistance in managing stress and depression. After 2 years of the pandemic, Indonesian senior high school teachers reported elevated levels of anxiety and somatic and social dysfunction [6].

In general, mental health services in Indonesia are limited, with psychologists, psychiatrists, and therapists being more accessible in major cities and not covered by national insurance [1-3]. There are significant disparities in the level of mental health literacy among those engaged in the health care system throughout various regions of Indonesia. The guidelines and allocation of support for mental health practitioners still present unresolved concerns. In certain regions of Indonesia, accessing the nearest community health clinics for mental health services can be challenging and costly due to a lack of competent and knowledgeable human resources in this field [7]. As a result, Indonesian senior high school teachers face challenges to treat the symptoms of mental disorders that arise during their working hours, because there are few public hospitals that have a psychology/psychiatry department [6,7]. Teachers can also get professional mental health services at a private hospital. However, private hospitals charge 4 times more than public hospitals for 1 psychotherapy season [6,7].

In view of the aforementioned circumstances, it is vital to equip schoolteachers in Indonesia with early interventions that are easily available, private, and affordable, and 1 feasible approach is to deploy a smartphone mobile app. A smartphone has multiple capabilities, such as presenting on-demand video resources and opportunities for live engagement with users. Consequently, it can be used to provide schoolteachers with accessible, inexpensive, and extended psychological interventions. Research has found that individuals with stress management skills and favorable living conditions are likely to have more long-term happiness [8]. Mindfulness-based interventions are 1 category of interventions that may be effective in improving well-being and stress management skills [9]. One popular mindfulness-based intervention is “Mindfulness-Based Stress Reduction” (MBSR). The MBSR was originally developed to address a variety of diseases in clinical and nonclinical populations. The program consists of 8 weekly sessions, plus approximately 45 minutes of daily home practice [10].

Much research has examined the effectiveness of the MBSR and other forms of mindfulness meditation in addressing mental health symptoms. Studies have revealed that the effect sizes for the MBSR are larger than those for other kinds of mindfulness meditation, likely due to the fact that the MBSR uses a variety of processes, such as formal meditation practices and live instruction by a trained facilitator [10-12]. Several studies have demonstrated the efficacy of mindfulness interventions for schoolteachers’ stress and anxiety [13-18], as well as stress related to COVID-19 [16]. However, relatively few studies have examined the effects of mindfulness training–based mobile apps for schoolteachers. One of the few empirical studies examined the effects of a 5-week standardized meditation program consisting of weekly offline classes and a suggested dose of 2 daily 20-minute home and school practice sessions [17]. The types of meditation practices included in this intervention included mantras and breath observation. The sample consisted of elementary, middle, and high school teachers who were randomly allocated to intervention (n=45) and control (n=46) groups. Findings indicated improvements in state and trait anxiety, reduction in emotional weariness (a component of burnout), and reduction in stress posttest, and lowered depersonalization (another component of burnout) at 1-month follow-up. Another study of the MBSR for primary school teachers [19] showed improvements in melancholy and stress, as well as increased tolerance and lowered prejudice (a component of the mindfulness scale) among schoolteachers following engagement in the intervention.

This feasibility study comprises 3 weeks of daily use of a mobile phone app. The lessons are organized based on components of the MBSR, and mobile apps are used to provide instructions for exercises requiring 10-20 minutes of daily home practice. Existing mindfulness interventions for general populations based on mobile apps for smartphones are primarily given in English, with rating stars based on user reviews [20]. There have been studies on the efficacy of mindfulness-based mobile apps in broad populations [20-22]. Several studies based in Iran have suggested the use of online and digital mindfulness programs as an efficient method to alleviate the stress related to COVID-19 and enhance psychological resilience [23]. To summarize, mindfulness-based mobile interventions have been demonstrated to be effective in reducing stress and enhancing well-being in a variety of settings [10,22,24].

To date, mobile app–based mindfulness interventions have not yet been implemented in the Indonesian language for senior high school teachers. According to the 2019 Global Economy Survey [5,6], which ranked Indonesia 72nd out of 97 countries in terms of the senior high school teacher-student ratio, there appears to be a decline in teacher supervision and student attention. As a result, schoolteachers are facing increased workloads, leading to higher levels of stress and physical complaints [4]. To support Indonesian senior high school teachers in managing anxiety and stress symptoms effectively and in a user-friendly manner, it is crucial to develop a brief mindfulness–based mobile app (BM-MA) intervention in Indonesia. Since such an app has not yet been implemented in Indonesia, this study aims to assess the feasibility and acceptability of the BM-MA to determine its suitability for further testing through a randomized controlled trial (RCT) [25].

### Study Objectives

This feasibility study will evaluate the possible adoption of the BM-MA in real-world contexts. The secondary objectives are to determine the effectiveness of BM interventions based on mobile apps in enhancing life satisfaction, self-compassion, and a sense of self-efficacy and in reducing participant-reported anxiety and stress symptoms. The following scientific questions will be addressed:

- Can the BM-MA be accepted into the community practice of Indonesian senior high school teachers to enable scale-up?
- Throughout the study, could the BM-MA be applied as intended?
- Could the BM-MA implementation be continued throughout the conclusion of the research?
- Does the BM-MA reduce stress and promote life satisfaction, self-compassion, and a sense of self-efficacy, as stated by participants?
- Does the BM-MA reduce participant-reported anxiety symptoms?

## Methods

### Overview of Study Design

This feasibility study will use a repeated-measures, between-group RCT design and adhere to the CONSORT (Consolidated Standards of Reporting Trials) statement (Multimedia Appendix 1). The feasibility protocol presented followed the SPIRIT (Standard Protocol Items: Recommendations for Interventional Trials) 2013 statement (Multimedia Appendix 2) [26]. As determined by an initial screening instrument, participants were only recruited if they exhibited good overall health, had no past experience with mindfulness practice, and had not recently experienced any adverse life events. Individuals who matched the inclusion criteria (explained further in the subsequent section) and responded within the recommended time frame of 2 weeks were routed to a webpage and a participant information sheet via the Qualtrics platform. Participants from 2 public senior high schools were informed that they would be randomly assigned to a treatment group or a wait-list control group (CG) and were asked to provide written informed consent (see Multimedia Appendix 3) prior to completing a brief demographic questionnaire. The participants were subsequently randomly assigned to either the BM-MA group or the CG. Participants in the CG were notified that they were on a waiting list and were asked to answer questionnaires at baseline, postintervention, and at 1-month follow-up before being allowed access to the BM-MA intervention for an additional 21 days (if they wished to use it).

At the end of the waiting period, the CG received thorough instructions regarding downloading and using the BM-MA intervention via a smartphone. In the meantime, the BM-MA group was instructed to complete questionnaires at baseline, postintervention, and at 1-month follow-up of the BM-MA intervention. After the baseline measures were completed, participants in the BM-MA group were provided with an access code and details of how to download the BM-MA to their smartphone. During the 21-day study period, they were urged to use the app daily. At baseline, postintervention, and at 1 month follow up of the BM-MA intervention, both groups were invited to attend a Zoom session to finish the measurements in real time, monitored by random assignments. Both groups were reminded about their right to withdraw from the study at any moment if they so chose. The follow-up evaluation was conducted 1 month postintervention for both groups. The intervention tries to ensure that no participant will face stigma as a result of participation by removing any potentially objectionable language according to the feedback from the local government partner, the Dinas Pendidikan Kepemudaan dan Olahraga Provinsi (Province Youth and Sports Office). The overview of the BM-MA pilot RCT study design following CONSORT guidelines is presented in Multimedia Appendix 1.

### Timeline and Study Setting

This feasibility study was conducted among schoolteachers from 2 public senior high schools in Indonesia over a 21-day period (Multimedia Appendix 4). A total of 30 participants per group (BM-MA and control) were adequate to establish feasibility [27]. A demographic survey of senior high school teachers in Indonesia [4] revealed that the median monthly pay for schoolteachers was IDR 5,000,000 (~US $321). Almost all the participants have a smartphone (n=55, 91.7%) and internet access on their smartphone (n=60, 100%), and only 17 (28%) said they had no intention of obtaining mental health treatment. To the best of our knowledge, there has been no formal record of senior high school teachers in Indonesia using mental health treatments. Nevertheless, the remarkable progress of Indonesia’s mental health care system is still restricted to a few major cities on Java Island [7].

### Local Stakeholder Partner

This study will be conducted in partnership with local education authorities in a number of Indonesian provinces, specifically the Dinas Pendidikan Kepemudaan dan Olahraga Provinsi (Province Youth and Sports Office). The government education authorities are responsible for the following:

- The creation of education-related technological policy
- The implementation of educational policy and the growth of language, literacy, and literature
- Education administration in the realm of education
- Implementation of program planning, assessment, and reporting
- The execution of other functions assigned by the governor that are linked to their duties and responsibilities

On the basis of the aforementioned capabilities, the local government partners made a major contribution to recruiting participants to take part in this feasibility study by sending invitations over email to prospective participants.

### Intervention

The BM-MA consists of audio guides of BM practices derived from mindfulness exercises available in the MBSR and mindfulness-based cognitive therapy (MBCT), an intervention adapted from the MBSR for preventing relapse in major depression. Examples of mindfulness practices that are included in the BM-MA are mindfulness of breathing, mindfulness of sounds, body scan meditation, mindful stretching, mountain meditation, and loving-kindness meditation. The audio guides for these mindfulness practices were developed (in the Indonesian language) by qualified MBCT teachers based in Indonesia and professionally recorded in an authorized recording studio. Each audio guide lasts between 5 and 20 minutes, and participants will be expected to engage in 10-20 minutes of mindfulness practice in total daily.

A progression of mindfulness practices was recommended to the participants in the first 10 days of practice, beginning with brief, introductory mindfulness exercises (eg, mindfulness of breathing), followed by practices that are lengthier and more open ended in nature (eg, mountain meditation, choice-less awareness meditation). After the first 10 days of practice, the participants were allowed to daily engage in any practice tracks that they like. The app also contains a feature that allows participants to track the overall duration and frequency of practice over a period of 21 days. Prior to downloading the app, the participants received an orientation to mindfulness practice by a trained mindfulness teacher via a Zoom meeting, and the session consisted of an introduction to the practice of mindfulness, stigma related to mental health, common myths about mindfulness practice, setting up a suitable environment for mindfulness practice, and troubleshooting potential difficulties that may arise during mindfulness practice (eg, dealing with difficult thoughts and emotions). The orientation session served the purpose of establishing a common level of mindfulness knowledge among the participants and ensuring their comprehension of how to use the BM-MA. They were asked to set aside time daily to commit to home mindfulness exercises using the app.

### Recruitment and Sample Size

This study’s sample comprised Indonesian high school teachers. The sample was drawn from 2 public senior high schools after a request for volunteers was sent over email by local government partners. Individuals who answered within 1 week were directed to a webpage on Qualtrics that describes the study and screening process. In addition, recruiting advertisements based on the opinion of the school principals were distributed via the schools’ social media. Individuals who passed the screening process were included in the participant pool.

The inclusion criteria for the feasibility study were Indonesian certified senior high school teachers (1) who are aged 18 years and older, (2) can read and write in Indonesian, (3) have access to a smartphone, and (3) have a total Generalized Anxiety Disorder-7 (GAD-7) score of no more than 14 and a total General Health Questionnaire-12 (GHQ-12) score of no more than 20. The exclusion criteria for the feasibility study were (1) regular practice of mindfulness meditation (eg, 15-20 minutes per day, 2-3 times per week), (2) a history of ongoing and present treatment for a mental disorder, (3) a positive response to GHQ-12 questions regarding suicidal ideation, and (4) refusal to provide written informed consent. These criteria were designated to recruit participants without prior regular experience in mindfulness practice and with no severe symptoms of depression and anxiety. The criteria also contributed to a more homogenous selection of participants across conditions.

Random sampling was used in the 2 public senior high schools in Indonesia, with the unit that was randomized being the senior high schools themselves. The sample size for this feasibility study was 30 participants for each study arm, with 95% CIs and small standardized effect sizes [27].

### Acceptability and Feasibility

A mixed methodological study was used to evaluate the BM-MA’s feasibility and acceptability. Acceptability is defined as the extent to which an app is suitable [28], and is assessed by analyzing participants’ qualitative responses to structured interviews. The quantitative scale used to measure feasibility was the Client Satisfaction Questionnaire-8 (CSQ-8) [28]. Each item in the CSQ-8 is rated on a scale of 1-4, and total scores range from 8 to 32. More than 70% of participants must receive a score of 24 or above on the CSQ-8 [28], given a 10% acceptable error rate [29], and participate in structured qualitative interviews for acceptance to be determined.

Feasibility is defined by Feeley et al [30] as the ease of deployment of an app among adults. In this study, on the basis of app scores, feasibility will be evaluated descriptively by examining the ease of recruitment, enrollment rate, and attrition rate. Participants will also be required to complete an in-person final evaluation. End-of-line evaluations will involve the viability and acceptance of the mobile app, as well as structures qualitative interviews.

### Randomization and Blinding

Participants from 2 public senior high schools were randomly assigned to the BM-MA group or the CG in a 1:1 ratio. The app arbitrarily selected a block size (2, 4, or 6) and assigned a random order within the block. For example, we randomly allocated 30 participants to each group in this scenario. The BM-MA group was denoted as “M” and the CG as “C.” The possible groupings were MCCM, MMCMCC, CM, MC, and CMCM. As part of the screening consent procedure, participants were informed that this will be an interventional study in which they might be randomized to a variety of BM-MA groups.

The prior online mindfulness intervention revealed a substantial potential for attrition. To proactively address this problem, we identified all potential avenues for recruiting and factored in the attrition rate when determining the appropriate sample size [27,30]. Considering the features and design of this pilot project, it would not be possible to blind the treatment assignment and result evaluation. However, participants were provided with information regarding the significance of a 2-arm study design following the CONSORT checklist (can be seen in Multimedia Appendix 1) and were strongly advised against disclosing their group allocation to their coworkers. Although there may be possible problems, this strong methodology will guarantee that the BM-MA intervention is carried out with a high level of academic excellence.

### Assessments Tools

There were 4 points of measurement: screening, baseline, end line, and follow-up. In Table 1, following the SPIRIT 2013 statement [26], an overview of the evaluation instruments used in this study is presented. Both groups received compensation for the time they spent and the internet data they used throughout the baseline, end line, and follow-up periods. The compensation amounts were ~US $3 for baseline, US $7 for the end line, and US$ 10 for follow-up, which is equivalent to the average cost of obtaining internet data in Indonesia. The act of providing monetary compensation as reimbursement is noncontroversial and commonly acknowledged [31].

**Table 1:** Study outcomes and time points of measurement.

Outcome	Instrument	Screening	Baseline	End line	Follow-up
			Day 1	Day 21	Day 41
Sociodemographics (age, education level, sex, marital status, occupation, years as schoolteacher, salary, schoolteacher status)	Demographics	Y^a^	Y	—^b^	—
Source of obtaining BM-MA^c^ information	Demographics	Y	—	—	—
Mental health care service history	Demographics	Y	Y	Y	Y
Smartphone ownership	Demographics	Y	Y	—	—
Internet access	Demographics	Y	Y	—	—
Contact details	Demographics	Y	—	—	—
Convenient contact times	Demographics	Y	—	—	—
Perceived stress	PSS^d^	—	Y	Y	Y
Depression symptoms	GHQ-12^e^	Y	Y	Y	Y
Anxiety symptoms	GAD-7^f^	Y	Y	Y	Y
Well-being	SLS^g^ and SCS-SF^h^	—	Y	Y	Y
Self-efficacy	TSES^i^	—	Y	Y	Y
Mindfulness trait	FFMQ^j^	—	Y	Y	Y
Engagement and experiences with the BM-MA	Questionnaires	—	Y	Y	Y
Qualitative interview of feasibility and acceptability	Interview guideline	—	—	Y	Y

^a^Y: measured.

^b^Not measured.

^c^BM-MA: brief mindfulness–based mobile app.

^d^PSS: Perceived Stress Scale.

^e^GHQ-12: General Health Questionnaire-12.

^f^GAD-7: Generalized Anxiety Disorder.

^g^SLS: Satisfaction With Life Scale.

^h^SCS-SF: Self-Compassion Scale—Short Form.

^i^TSES: Teachers’ Sense of Efficacy Scale.

^j^FFMQ: Five Facet Mindfulness Questionnaire.

#### Sociodemographic and Personal Information

During the screening session, participants’ sociodemographic and personal information was collected using a combination of questions. The open questions inquired about participant age, years as a teacher, and salary, whereas the closed questions required participants to select from a list of options, such as the education level and internet access on a smartphone (yes/no). To ensure participants’ eligibility, sociodemographic and personal data were requested once again at baseline.

#### Measurement of Primary Outcome

The evaluation of the BM-MA implementation in real-world contexts will be the primary outcome for this study. This study will evaluate the adoption of the BM-MA with an engagement and experience questionnaire. On the basis of Howell et al’s [22] findings, a questionnaire will be designed to allow participants to rate their experience using the app.

#### Measurement of Secondary Outcomes

Anxiety was measured with the GAD-7 as a secondary outcome. The GAD-7 is a brief, self-administered scale with a few measures for measuring anxiety that was first administered to primary care patients [32,33]. The scale has been translated into other languages. People have adjusted the items to suit different contexts. It has high psychometric qualities, which enables it to detect anxiety and depression quickly and precisely [32]. Items are rated from 0 (absence of anxiety) to 3 (anxiety is felt most of the days) [33], and the overall score ranges from 0 to 21. Higher scores indicate that the respondent exhibits more anxiety symptoms. In this study, the GAD-7 demonstrated strong reliability and validity in terms of criteria, the concept, and factorial and procedural measures. An optimal cut-off was set that maximized sensitivity (89%) and specificity (82%) [32].

To determine the psychological well-being of participants, we measured their life satisfaction, sense of self-efficacy, and self-compassion. Life satisfaction was measured with the Satisfaction With Life Scale (SLS). Perceived self-efficacy was measured with the Teachers’ Sense of Efficacy Scale (TSES) [34]. For the TSES, higher scores show a higher sense of self-efficacy. The reliability of the TSES subscales was 0.91 for instruction, 0.90 for management, and 0.87 for engagement. The intercorrelation among the instruction, management, and engagement subscales was 0.60, 0.70, and 0.58, respectively, with a significance level of *P*<.001 [34]. Self-compassion was measured with the 12-item Self-Compassion Survey—Short Form (SCS-SF) [35]. The SCS-SF includes 3 subscales: self-kindness versus self-judgment, common humanity versus isolation, and mindfulness versus overidentification. Higher scores indicate higher self-compassion. The internal consistency of the SCS-SF was found to be satisfactory, with Cronbach α of .86 or higher in all samples [35].

In addition, depression symptoms of the participants were measured using the GHQ-12. This scale has been psychometrically validated for use as a screening instrument for depression symptoms in primary health services in numerous countries and has high validity and reliability [36]. Reliability analysis revealed a respectable outcome, with Cronbach α of .87 [37]. The most common scoring methods are bimodal (0-0-1-1) and Likert scoring styles (0,1,2,3) [37]. For the GHQ-12, higher scores indicate depression and physical and social dysfunction. The perceived stress condition was assessed using the 10-item Perceived Stress Scale (PSS) [38]. This scale examines the responses of participants to a variety of potentially stressful circumstances with 2 variables. The reliability coefficients for the scores on these 2 variables were 0.87 and 0.73, respectively [38]. Higher scores indicate a higher level of perceived stress.

The mindfulness trait was assessed using the Five Facet Mindfulness Questionnaire (FFMQ). This instrument was created on the basis of a factor analytic assessment of 5 independently developed mindfulness questionnaires [39]. The FFMQ has repeatedly demonstrated strong psychometric qualities, including high levels of construct validity and reliability. The subscales of the FFMQ have Cronbach α ranging from 0.73 to 0.91, indicating good internal consistency [40]. Baer et al [39] identified 5 parameters that appear to represent mindfulness aspects as they are currently conceived: observing, describing, acting with awareness, not judging the inner experience, and not reacting to the inner experience. Higher scores indicate a greater level of mindfulness in individuals.

### Ethical Considerations

#### Ethical Approval

The BM-MA is a digital nonpharmaceutical mental health intervention whose content was adapted from 1 of the existing mindfulness-based apps that has been shown to be safe for treating anxiety [41]. The trial protocol was approved by the Research Ethics Committee at Universitas Pendidikan Ganesha (1828/UN48.16/LT/2022) and publicly registered (Chinese Clinical Trial Registry with protocol number ChiCTR2300068085). The intervention also ensured that no participant will be stigmatized for their participation. An introduction meeting was held on Zoom to address the potential stigma around mental health interventions and explore strategies for effectively managing this stigma. We will be communicating to the research committee in the case of any important protocol modifications. Written informed consent was obtained from each participant. The intervention content, which includes an activity to learn and practice mindfulness exercises via a mobile app, was rewritten or sensitive or potentially objectionable terms were eliminated.

#### Data Management

Data collection was conducted electronically. The data were securely stored on password-protected computers, and only members of the study group have access to them. To ensure the confidentiality of the participants, distinct, unique study numbers were used for data storage, tracking, and reporting. An encryption protocol used complex algorithms to obscure data, which it then decoded using an encryption key that the interaction’s sender provided. The data cleaning procedure systematically and proactively identified mistakes. Errors were deliberately and methodically sought out through the data cleaning procedure. Repeated imputation was used to manage missing data due to its reliability in dealing with missing data in a feasibility study and its availability for the vast majority of data types [42].

### Data Analyses

The sociodemographic characteristics of the participants (eg, age) will be analyzed descriptively. Missing data, including participants who dropped out, will be treated using the intention-to-treat principle with multiple imputations of missing values [42]. Intention-to-treat analysis is a statistical method used in prospective randomized studies. It involves including all individuals who were randomly assigned to a group, regardless of whether they received any sort of treatment. These participants are then examined on the basis of their initial group assignment. This methodology enables the researcher to derive precise and impartial judgments regarding the efficacy of an intervention. This approach maintains the advantages of randomization, which cannot be presumed when using alternative methods of analysis.

The data will be examined using repeated-measures ANOVA with groups (BM-MA vs control) and 3 time periods as factors (baseline, day 21, day 41). In addition, Pearson correlation analysis will be undertaken to determine whether the subjective ratings of involvement and experience with the BM-MA by participants correlate with any potential improvements in well-being experienced by the BM-MA group. The variation in findings between the 2 groups will be encompassed by the 95% CI range for the significance of the effect. The amount of effect (Cohen d) will be calculated by dividing the median variation (from baseline, monitoring, and end line to follow-up) obtained from the results of the linear mixed model by the pooled SD at baseline.

Transcription will be conducted for all records of structured qualitative interviews. The qualitative data will undergo deductive theme analysis using a framework and will be used to create an initial codebook. The qualitative data will be analyzed using an iterative method by 2 coders, who will independently code all transcripts and then discuss any inconsistencies until consensus is established. The resulting comprehensive codebook will be input into qualitative analysis software.

## Results

**Figure 1:**
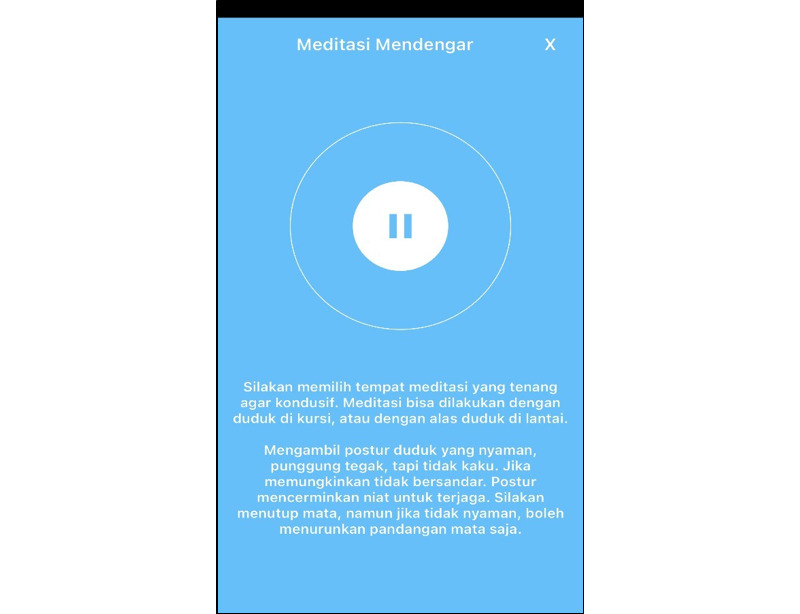
Screenshot 1 of the brief mindfulness–based mobile app (BM-MA).

**Figure 2:**
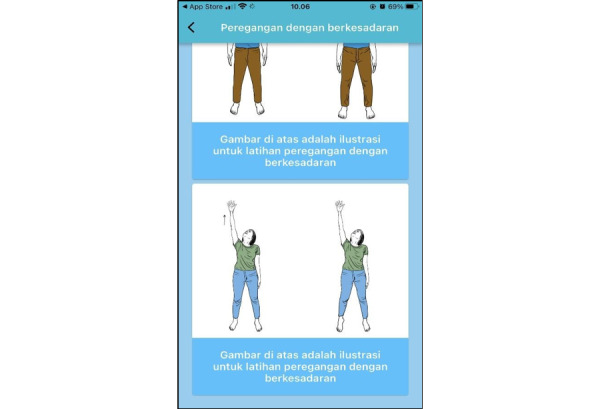
Screenshot 2 of the brief mindfulness–based mobile app (BM-MA).

**Figure 3:**
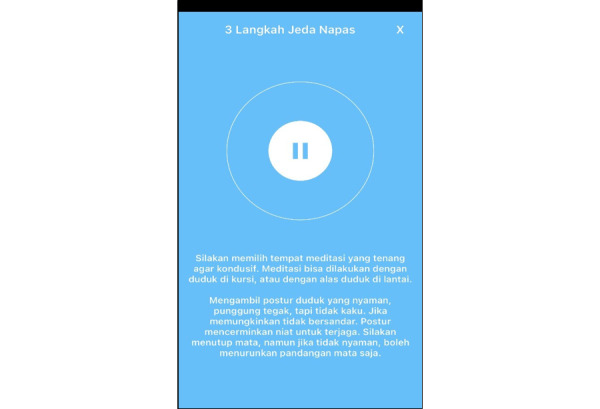
Screenshot 3 of the brief mindfulness–based mobile app (BM-MA).

This protocol funding started in May 2023. Participants for this pilot RCT were recruited in December 2023, and the RCT was conducted from January through March 2024. Data analysis was conducted from March through May 2024. Paper writing based on the data analysis is ongoing. The expected results of this study will be published in December 2024. The BM-MA display can be seen in Figures 1-3.

## Discussion

### Summary of Findings

This study seeks to explore the feasibility, acceptability, and efficacy of the BM-MA for Indonesian high school teachers. According to our understanding, the BM-MA is the first evidence-based nonpharmacological intervention in the Indonesian language aimed toward communities, particularly senior high school teachers, in order to address the difficulties in accessing mental health treatments in Indonesia. This protocol provides exhaustive details on how the intervention will be carried out. The reason for the study design, including its overview and context, has been provided.

This protocol offered extensive information regarding the execution of the trial. The study design’s explanation has been clarified, comprising its overview and setting. Furthermore, theoretical and pragmatic reasons for the implementation of recruitment, screening, intervention, and assessment have been acknowledged, including primary and secondary assessments, as well as follow-up evaluation. Efforts have been made to address both ethical and safety concerns in order to uphold the integrity of the research and ensure the safety of the participants. The protocol implementation has been additionally enhanced by the participation of teacher communities in recruitment and the significant degree of enthusiasm among participants in digital mental health interventions.

The BM-MA intervention feasibility protocol will investigate multiple outcomes, such as teachers’ life satisfaction, self-efficacy, self-compassion, anxiety, stress, and mindfulness state, among senior high school teachers in Indonesia. The outcome of this feasibility study will also examine pre- and postintervention measures to detect any significant changes. The consequences of the findings will determine whether a BM-MA intervention should be recommended for efficacy testing [25]. This feasibility study aims to assess the potential implementation of the BM-MA in practical applications.

Nonetheless, any trial may encounter difficulties, particularly with attrition, a typical issue in clinical trials [43]. To anticipate the problem of attrition, all teacher communities, including the local government partner (Dinas Pendidikan Kepemudaan dan Olahraga Provinsi, Province Youth and Sports Office), that contribute to the recruitment process were discovered and the rate of attrition accounted for in the sample size calculation [43]. The participants were provided with an explanation of the relevance of the study. Attrition can be solved with the condition that 72% of participants in the preliminary survey [6] had a strong interest in using digital mental health interventions.

The study findings have practical implications for the adoption of digital mental health interventions in Indonesia, particularly among schoolteachers. The preparedness of the BM-MA to be publicly accessible, in partnership with local government partners in the education sector, is a key factor. Undoubtedly, the potential impact of the BM-MA in addressing the mental health needs of schoolteachers is highly important. Therefore, it is imperative to consider collaborating with the Indonesian government to enhance treatments.

The potential future research directions based on the study findings could involve performing larger-scale efficacy trials or investigating the long-term benefits of the BM-MA for the wellness of schoolteachers. It is necessary to evaluate potential areas for improving or adapting the intervention based on feedback from participants and recent research, such as integrating functionalities within the app to guarantee its accessibility for users with specific requirements.

Hence, we believe that the BM-MA intervention passes rigorous academic criteria. We will publish the findings of the feasibility study in both an international journal and an international conference. We will use the Contributor Roles Taxonomy (CRediT) to evaluate the authorship and provide a detailed description of each author’s data set and statistical code.

### Conclusion

This study will establish essential information regarding the feasibility and efficacy of the BM-MA, a digital mental health intervention modified from 1 of the existing mindfulness-based apps, as well as its scalability. This research is crucial for developing knowledge and practice in the field of digital mental health. If the BM-MA demonstrates a considerable positive effect on life satisfaction, self-efficacy, self-compassion, anxiety, and stress among Indonesian senior high school teachers, it will have the potential to be used by Indonesian schoolteachers who might also develop anxiety symptoms and find it challenging to seek expert mental health services. This feasibility study’s findings will be published in an international journal and also presented at a conference. Moreover, the future application the BM-MA could be through collaborative efforts with the Indonesian government.

## Appendix

Multimedia Appendix 1 CONSORT (Consolidated Standards of Reporting Trials) checklist.

Multimedia Appendix 2 SPIRIT (Standard Protocol Items: Recommendations for Interventional Trials) checklist.

Multimedia Appendix 3 Informed consent.

Multimedia Appendix 4 Figure S1. Overview of BM-MA pilot randomized controlled trial study design following CONSORT diagram. BM-MA: brief mindfulness–based mobile app; CONSORT: Consolidated Standards of Reporting Trials.

## Abbreviations

BM: brief mindfulness
BM-MA: brief mindfulness–based mobile app
CG: control group
CONSORT: Consolidated Standards of Reporting Trials
CSQ-8: Client Satisfaction Questionnaire-8
FFMQ: Five Facet Mindfulness Questionnaire
GAD-7: Generalized Anxiety Disorder-7
GHQ-12: General Health Questionnaire-12
MBCT: mindfulness-based cognitive therapy
MBSR: Mindfulness-Based Stress Reduction
PSS: Perceived Stress Scale
RCT: randomized controlled trial
SCS-SF: Self-Compassion Scale—Short-Form
SLS: Satisfaction with Life Scale
SPIRIT: Standard Protocol Items: Recommendations for Interventional Trials
TSES: Teachers’ Sense of Efficacy Scale
