# Supplementary material for: Brief Mobile App–Based Mindfulness Intervention for Indonesian Senior High School Teachers: Protocol for a Pilot Randomized Controlled Trial
Source: JMIR Res Protoc. 2024 Oct 23;13:e56693. doi: 10.2196/56693 (PMC11541156; doi:10.2196/56693)
Supplement: Multimedia Appendix 3 [file resprot_v13i1e56693_app3.docx]

**Chinese Clinical Trial Register
Adult Informed Consent Form (online intervention)**

My name is Dr. XXX. I am an Assistant professor in XXX. Together with the village authorities in Bali province, we are developing an electronic intervention that can help improving psychological well-being for early adults, and we are planning to try it out, which we invite you to take part in. You are being invited to participate in this study because you are early adults who live in Balinese rural areas.

**Aim**

The purpose of this study is to examine the feasibility, acceptability and effectiveness of The Brief Cognitive Behavioural Therapy based on mobile apps (MB-CBT) for early adults in Bali rural areas.

**Procedures**

If you agree to be involved in this study, we will ask you to complete an online survey, which will collect basic demographic information and help us decide whether you meet the criteria to be part of the study. If you are eligible, you will be given access to an online program, which will teach you techniques of self-management.

You will be randomly assigned either to an experimental group or to a ‘wait-list’ control group and will be asked to provide informed consent before completing a brief demographics questionnaire. You will then be allocated to the MB-CBT or to a control group at random. The control group will be informed that they are a ‘wait-list’ group, and will be required to complete questionnaire measures at baseline, 16, and 30 days later, before being offered access to the MB-CBT intervention for a further 16 days (should they wish to use it). The wait-list group or the control group will be given detailed instructions of how to download and use the MB-CBT intervention via their smart phone at the end of the wait-list period. Meanwhile the treatment group will be given instructions to complete questionnaire measures at baseline and then again at 16, 30 and 46 days of the MB-CBT intervention. After the completion of the baseline measures, the participants in the treatment group will be given an access code and details of how to download the MB-CBT programme to their smart phone. You will be encouraged to use the app every day during the 16-days study period, and to keep a diary to record hours/days of activity/usage. Email will be sent out within 24 hours of completing the full 16-days intervention to remain you to complete all outcome measures as end line assessment. If no action is taken further email reminders will be sent within 10 days of completion. Participants in both groups will be reminded that they have the right to withdraw at any point during the study if they wish to do so. The follow-up assessment will be done for both groups on 16 days after end line assessment.

**Study location**: All study procedures will take place online.

**Benefits**

Your involvement in this study can potentially improve your psychological well-being. Information gain from this study will also provide insights into ways to reach out and engage early adults in Balinese rural areas, as well as improving the electronic intervention which will be delivered to a wider range of early adults in Indonesian rural areas in the future.

**Risks**

There are no anticipated risks associated with the study. However, if you do participate and find that you are uncomfortable to any of the content of the online sessions or questionnaire items, you are free to withdraw from this study without any penalty.

**Confidentiality**

All data in the study will be collected electronically and will be held securely on password-protected computers that can be accessed only by the members of the research group. To protect participant confidentiality, unique anonymous study IDs will be used for data storing, tracking, and reporting.

*Retaining research data:* When the research is completed, I may save the data for use in future research done by others or myself. I will retain this study information for up to 5 years after the collection.
