# Supplementary material for: Brief Mobile App–Based Mindfulness Intervention for Indonesian Senior High School Teachers: Protocol for a Pilot Randomized Controlled Trial
Source: JMIR Res Protoc. 2024 Oct 23;13:e56693. doi: 10.2196/56693 (PMC11541156; doi:10.2196/56693)
Supplement: Multimedia Appendix 4 [file resprot_v13i1e56693_app4.docx]

Figure 1. Overview of BM-MA pilot RCT study design following CONSORT diagram

Preparation

Recruitment in 2 public senior high school (N=75)

Screening (N=72)

BM-MA development and internal testing

Contact to community partner and stakeholder

Socio-demographic

GAD-7 and GHQ-12

Assessment, allocation, and treatment

Baseline assessments (N=66)

Decline to participate (N=2)

Control group (N=32)

Intervention group (N=32)

Day 1-21 practices

End line assessments

Local community partner and collaboration with researchers

“Adoption” BM

Day 1-21 practices

Attrition (N=3)

Not eligible (N=6)

Follow-up

“Maintenance”

(BM-MA)

Follow-up assessments BM-MA

Adoption (BM-MA)

“Implementation” (BM-MA)

Intervention group (N=32)

Randomization

Control group (N=32)
